# Barriers and Enablers Influencing Dietetic Practice in the Management of Polyendocrine Metabolic Ovarian Syndrome

**DOI:** 10.3390/nu18183010

**Published:** 2026-09-14

**Authors:** Jaye Lange, Nicole Scannell, Evangeline Mantzioris, Lisa Moran, Anthony Villani

**Affiliations:** 1 School of Health, University of the Sunshine Coast, Sippy Downs, QLD 4556, Australia; jayelange@gmail.com (J.L.); nicole.scannell@research.usc.edu.au (N.S.); 2 Alliance for Research in Exercise, Nutrition and Activity (ARENA), College of Health, Adelaide University, Adelaide, SA 5000, Australia; evangeline.mantzioris@adelaide.edu.au; 3 Monash Centre for Health Research and Implementation (MCHRI), School of Public Health and Preventive Medicine, Monash University, Melbourne, VIC 3800, Australia; lisa.moran@monash.edu; 4 School of Allied Health and Human Performance, College of Health, Adelaide University, Adelaide, SA 5000, Australia

**Keywords:** polyendocrine metabolic ovarian syndrome, dietitians, knowledge, confidence, dietary management

## Abstract

Background/Objectives: Polyendocrine Metabolic Ovarian Syndrome (PMOS) is a common endocrine disorder affecting women of reproductive age. Current international evidence-based guidelines promote lifestyle management, including diet and nutrition, as first-line treatment strategies. Accredited nutrition professionals, such as dietitians, are therefore well placed to deliver such support. We aimed to investigate the barriers, facilitators and practice patterns of accredited dietetic professionals in the management of PMOS. Methods: This was a mixed-methods, cross-sectional analysis of Australian dietitians using a self-administered online survey to collect: self-rated knowledge and confidence levels for PMOS care; perceived barriers and facilitators to clinical management; awareness and implementation of evidence-based guidelines; and current dietary management practices. Quantitative and qualitative data were analyzed in parallel, using both descriptive statistics and conventional content analysis. Results: Dietitians (n = 86) reported moderate levels of knowledge (n = 33; 38.4%) and confidence (n = 31; 36.5%). Perceived practice barriers included supporting women through weight management, lack of interprofessional collaboration and dietary misinformation. Facilitators to clinical management included the availability of evidence-based dietary approaches, professional development opportunities and clinical knowledge and experience. Manipulation of macronutrients, dietary patterns, individualized medical nutrition therapy and lifestyle and eating behaviors were identified as key dietary management approaches. Conclusions: Moderate levels of knowledge and confidence were observed. However, ongoing barriers, including supporting women through weight management strategies, limited interprofessional collaboration and dietary misinformation, continue to contribute to inconsistent practice patterns. Our findings highlight the need for structured professional development and improved implementation of evidence-based PMOS guidelines in dietetic practice.

## 1. Introduction

Polycystic ovary syndrome (PCOS), recently renamed Polyendocrine Metabolic Ovarian Syndrome (PMOS) [1], is the most common endocrine disorder affecting women of reproductive age, with heterogeneous clinical features across multiple body systems [2,3]. Globally, the prevalence of PMOS affects approximately 12% of women, depending on the population studied and the criteria employed [4,5]. PMOS is a complex and multifaceted condition with key reproductive, metabolic, and psychological features [3,6]. Intrinsic insulin resistance, a key characteristic of the pathophysiology of PMOS, is mechanistically distinct from that associated with excess weight or adverse lifestyle factors [7]. However, extrinsic insulin resistance arising from excess body weight further worsens reproductive, metabolic and psychological symptoms [8,9]. Women with PMOS are biologically predisposed to have greater weight gain and exhibit a higher prevalence of overweight and obesity, which compound these pathophysiological mechanisms [9]. Moreover, a greater understanding of the heterogeneity of the pathophysiology of PMOS may facilitate an improvement in existing therapeutic interventions, or indeed promote the need for novel management strategies, including diet and lifestyle. For example, Szczuko et al. [10] reported differences in phenotypic characteristics in women with PMOS, particularly in relation to differences in the concentration between tumor necrosis factor (TNF-α) and insulin-like growth factor I (IGF-I) and their association with androgen levels [10].

Given the diverse symptom profile, PMOS management therefore requires a multidisciplinary approach [3]. The current evidence-based guidelines [11] advocate for a comprehensive, long-term model of care encompassing cardiometabolic, dermatological, and psychological screenings, ongoing risk education, lifestyle management, and reproductive monitoring. Despite these recommendations, many women report dissatisfaction with care and inadequate support, particularly regarding lifestyle and dietary management [12,13,14]. Furthermore, access to dietetic support appears limited, with few women receiving referrals to dietitians for dietary management, suggesting that some primary healthcare providers may lack confidence in the effectiveness and feasibility of lifestyle interventions for PMOS [15].

Maintaining a healthy lifestyle, including sustainable healthy eating, physical activity, and weight management, including weight gain prevention, weight loss and weight loss maintenance, is integral to the long-term management of PMOS [16]. Nutrition professionals, such as dietitians, therefore have the potential to play a pivotal role in supporting the lifelong health and well-being of women with PMOS [17]. Although numerous dietary interventions for PMOS have been investigated [18,19,20], there is limited high-quality evidence favoring any single dietary approach [11]. Consequently, current evidence-based guidelines [11] are consistent with general population recommendations, focusing on healthy lifestyle modification consistent with population-based dietary guidelines [21]. Despite these recommendations, there remains limited research exploring which nutritional strategies Australian dietitians are currently employing in practice to assist women with PMOS [22]. As such, understanding the practice patterns of dietitians may aid in identifying important practice-related gaps to inform and target future professional development opportunities in order to upskill the current and future workforce.

Therefore, this study aimed to identify the barriers, facilitators and practice patterns, including knowledge, skills, confidence and dietary management strategies of Australian dietitians in the management of PMOS.

## 2. Materials and Methods

### 2.1. Study Design, Setting and Participants

This study utilized an online cross-sectional survey targeting Australian dietitians and employed a mixed-methodological study design. We adopted this methodological approach as it allowed us to capture both qualitative and quantitative data across a diverse range of dietetic practitioners in numerous practice settings throughout Australia. Eligible participants were all Accredited Practicing Dietitians (APDs) and/or advanced APDs (irrespective of their experience in PMOS management), who were dietetic professionals currently practicing in Australia and able to complete the online survey independently. No additional exclusion criteria were applied. The study was created and administered online through Qualtrics^TM^ survey software and included the study protocol and contact details of the research team.

Recruitment occurred between May and August 2025. A recruitment flyer was created and shared online via social media platforms such as and LinkedIn, as well as professional organizations and online platforms for nutrition professionals, including Dietitian Connection and Dietitians Australia. The flyer was also emailed to private dietetic practices around Australia. Given that the formal name change (from PCOS to PMOS) occurred following completion of the present study, for accuracy, we have maintained reference to PCOS in the presentation of the questionnaire and study results where relevant.

### 2.2. Data Collection

An 18-item self-administered online survey instrument (Table 1) was designed to collect both qualitative and quantitative data regarding APDs’ current knowledge, confidence and management strategies of PMOS. Data was collected using a combination of ordinal categorical items, nominal categorical multiple-response items, binary nominal items, Likert scale items and open-ended free-text questions. Given the lack of a previously validated survey instrument, and to determine face validity, the authors developed a prototype survey that was piloted against a representative sample of Australian dietitians. This assessment included the voluntary participation of a convenience sample aged between 32–48 years, who were representative of the current workforce of dietitians in Australia, practicing across a broad range of practice settings including clinical (n = 4), private practice (n = 3), community public health (n = 1), industry (n = 1) and research (n = 1). A total of n = 10 participants (all female, recruited from Southeast Queensland, New South Wales and Victoria) completed the prototype survey. No changes to the survey instrument were required during the piloting process. The questionnaire was divided into two distinct sections: (1) demographic and practice-related information; (2) knowledge, confidence and practice-related behaviors related to the dietary management of PMOS. Questionnaire items related to knowledge, confidence and practice-related behaviours were informed by previously published literature amongst dietitians and allied health professionals [22].

Respondents could select or submit multiple responses for questions related to: dietetic practice domains; areas of professional development; barriers to care; enablers of care; reasons for referrals; priority treatment areas; dietary management principles; and dietary approach rationale. The questionnaire was designed to take ~10 min to complete; however, no time restriction was imposed. Progression through the survey did not require responses to all preceding questions. Each survey link was uniquely identifiable by the distribution server to prevent duplicate responses. In addition, the investigators screened all participant responses, cross-checking IP addresses and postcodes, to ensure consistency with eligibility criteria. Responses violating the eligibility criteria for the present study were identified and excluded from all analyses.

### 2.3. Statistical Analysis

Quantitative data from the online questionnaire were analyzed using Statistical Package for the Social Sciences (SPSS) 31.0 for Windows software (IBM Corp., Armonk, NY, USA). Descriptive statistics were used to summarize participant demographics, experience with PMOS patients, professional development, knowledge and confidence levels, patient reasons for dietitian engagement and priority treatment areas. Continuous variables are presented as mean ± standard deviation (SD), and categorical variables are presented as frequencies or percentages.

Qualitative data were analyzed using conventional content analysis [23], whereby the data were read on two separate occasions to facilitate familiarization and identify initial codes. A recursive process was undertaken independently by two authors (JL and AV) during content analysis to preserve the rich detail and nuance of the data [24]. Open coding was applied to identify and group related codes into categories, which were subsequently applied to the full dataset and arranged within a framework matrix to facilitate theme development. Memos were employed throughout the analysis process to facilitate the organisation and understanding of the data and to guide coding and categorization. The analysis was led by one researcher (JL), with consensus checks undertaken by a second investigator (AV), who independently analyzed 100% of the data. The addition of a third researcher (EM) was implemented to resolve any discrepancies in open coding. Representative quotations illustrating the themes are presented alongside each theme and referenced with the participants’ number in brackets. Identified themes were counted for frequency and percentage responses.

## 3. Results

### 3.1. Participant Characteristics

A total of n = 95 APDs commenced the online survey, of which n = 86 (91%) completed all components of the questionnaire and were included in the final analysis. All responses to the questionnaire were included in the final analysis. Demographic characteristics are presented in Table 2. Participants were predominantly qualified at the bachelor’s (n = 41; 47.7%) or master’s level (n = 38; 44.2%), respectively. Participants reported a range of practice experience, including 1–5 years (n = 31; 36.0%), 6–10 years (n = 19; 22.1%), 10–20 years (n = 26; 30.2%) and more than 20 years (n = 10; 11.6%). The most common practice domain included private practice (n = 51; 46.3%), clinical or acute care (n = 23; 20.9%) and community settings (n = 18; 16.4%). Most participants reported previous experience providing dietary services and management for women with PMOS (n = 74; 86.1%). Just over two-thirds of participants reported engaging in PMOS-related PD (n = 60; 69.8%), with the most common reported activities being webinars (n = 17; 40.5%), self-study (n = 10; 23.8%) and formal courses (n = 8; 19.0%).

### 3.2. Knowledge and Confidence

Most participants responded to the knowledge (n = 86) and confidence (n = 85) items. Participants self-reported varying levels of knowledge in relation to dietary management principles of PMOS, with one-third of participants reporting ‘moderate’ levels of knowledge (very high: n = 13; 15.1%; high: n = 22; 25.6%; moderate: n = 33; 38.4%; low: n = 17; 19.8%). Only one participant reported having ‘none’ or ‘limited’ knowledge. Confidence in dietary treatment and management of patients with PMOS followed a similar pattern to self-reported knowledge (very high: n = 13; 15.3%; high: n = 22; 25.9%; moderate: n = 31; 36.5%; low: n = 18; 21.2%). Similar to the foregoing response, one participant self-rated their confidence as ‘none’ or ‘limited’ in this area.

### 3.3. Barriers and Facilitators to Dietary Management of PMOS

Among participants who completed the questionnaire, n = 115 barrier items were reported and grouped into common themes (Table 3). The most frequently cited barrier was weight management (n = 24; 20.9%). Specifically, this included difficulty in supporting women to achieve and maintain weight loss, despite the implementation of targeted lifestyle changes.

Dietitians also identified a lack of interprofessional collaboration (n = 19; 16.5%) between healthcare practitioners as a key barrier in the provision of care in patients with PMOS. Participants specifically noted difficulties engaging with professionals from other disciplines, including general practitioners (GPs), obstetricians and gynecologists and a lack of referrals from primary healthcare providers.

Dietary misinformation was also identified as an important barrier (n = 14; 12.2%) by dietitians. Respondents highlighted the influence of non-evidence-based sources of dietary information from social media and online platforms, which hindered their ability to facilitate behavior change. This often led to patients forming misconceptions about dietary approaches for the management of PMOS, such as ‘gluten-free’, ‘dairy-free’, ‘ketogenic’ and ‘paleo’ diets. Moreover, dietitians reported that the lack of specific and consistent evidence to guide dietary management in women with PMOS contributes to the spread of misinformation, including among other healthcare professionals.

Other less frequently reported barriers included individual symptoms and comorbidities; disordered eating behaviors; lack of knowledge and professional development opportunities; patient knowledge and expectations; patient motivation; hormonal dysfunction; pharmaceuticals, nutraceuticals and dietary supplementation; and food beliefs (Table 3).

In contrast, among respondents who completed the questionnaire, n = 101 facilitator items were identified and grouped into common themes (Table 3). Dietitians most frequently reported the application of evidence-based dietary approaches for the therapeutic dietary management of PMOS (n = 22, 21.8%). Dietitians described using previously established approaches with strong evidence in other similar populations, including general healthy eating principles, Mediterranean-style diets and adaptations from diabetic education. Of note, dietitians reported confidence in practice when applying and translating evidence-based strategies. The use of current research and guidelines gave them greater confidence in practice.

Opportunities for professional development were reported as another key facilitator by dietitians (n = 21; 20.8%). Respondents highlighted engagement in structured professional development and education modules, including nutrition for fertility and pregnancy as well as eating disorders. Self-directed learning was also reported, including reading current research, listening to podcasts, and following experts in the field. Additionally, greater visibility of PMOS management through social media was perceived as a valuable source of ongoing education and awareness.

Clinical knowledge and experience were also identified as a key enabler by dietitians (n = 20; 19.8%). Participants noted that specialized knowledge and prior clinical experience enabled them to individualize dietary management for women with PMOS by tailoring strategies to patient needs, applying behavioral-change techniques such as goal setting and utilizing educational resources. An in-depth knowledge of the pathophysiology of PMOS was also viewed as favorable, particularly regarding the diet-disease relationship. Other less frequently reported enablers included interprofessional collaboration, access to patient resources, behavior change, empathy and counselling skills and the use of pharmaceuticals, nutraceuticals and dietary supplements (Table 3).

### 3.4. Clinical Priority Areas

Dietitians provided n = 202 responses describing reasons women with PMOS engage with dietetic services, with many responses overlapping across similar themes. Weight loss (n = 66; 32.7%), followed by symptom management (n = 38; 18.8%), infertility (n = 36; 17.8%) and risk of T2DM (n = 27; 13.4%), were among the most frequently reported. Less frequently noted reasons were psychological health (n = 8; 4%), CVD risk (n = 7; 3.5%) and disordered eating (n = 7; 3.5%). When dietitians were asked to prioritize nutritional problems, out of the 312 responses, symptom management (n = 66; 21.2%), followed by infertility (n = 59; 18.9%) and weight loss (n = 57; 18.3%), were identified as the highest priorities. Diabetes risk (n = 48; 15.4%), psychological health (n = 45; 14.4%) and CVD risk (n = 33; 10.6%) were also identified as key nutritional problems, with disordered eating behaviours (n = 3; 1%) less frequently identified.

### 3.5. Use of Evidence-Based Guidelines for PMOS Management

A total of n = 44 (51.2%) respondents reported being familiar with evidence-based guidelines; however, of these respondents, one-quarter (n = 11; 25%) were able to identify the most recent iteration of the International Evidence-based Guidelines. Few respondents (n = 2; 2.3%) also referred to evidence-based resources such as those from Dietitians Australia, Practice-based Evidence in Nutrition, PubMed and Evidence-Informed Nutrition Network. Others described specific nutrition frameworks (n = 7; 8.1%), including recommendations related to lifestyle management such as healthy eating and exercise. Almost half of all respondents were not familiar with the evidence-based guidelines (n = 41; 47.7%).

### 3.6. Use of Dietary Management Approaches in Practice

**Figure 1:**
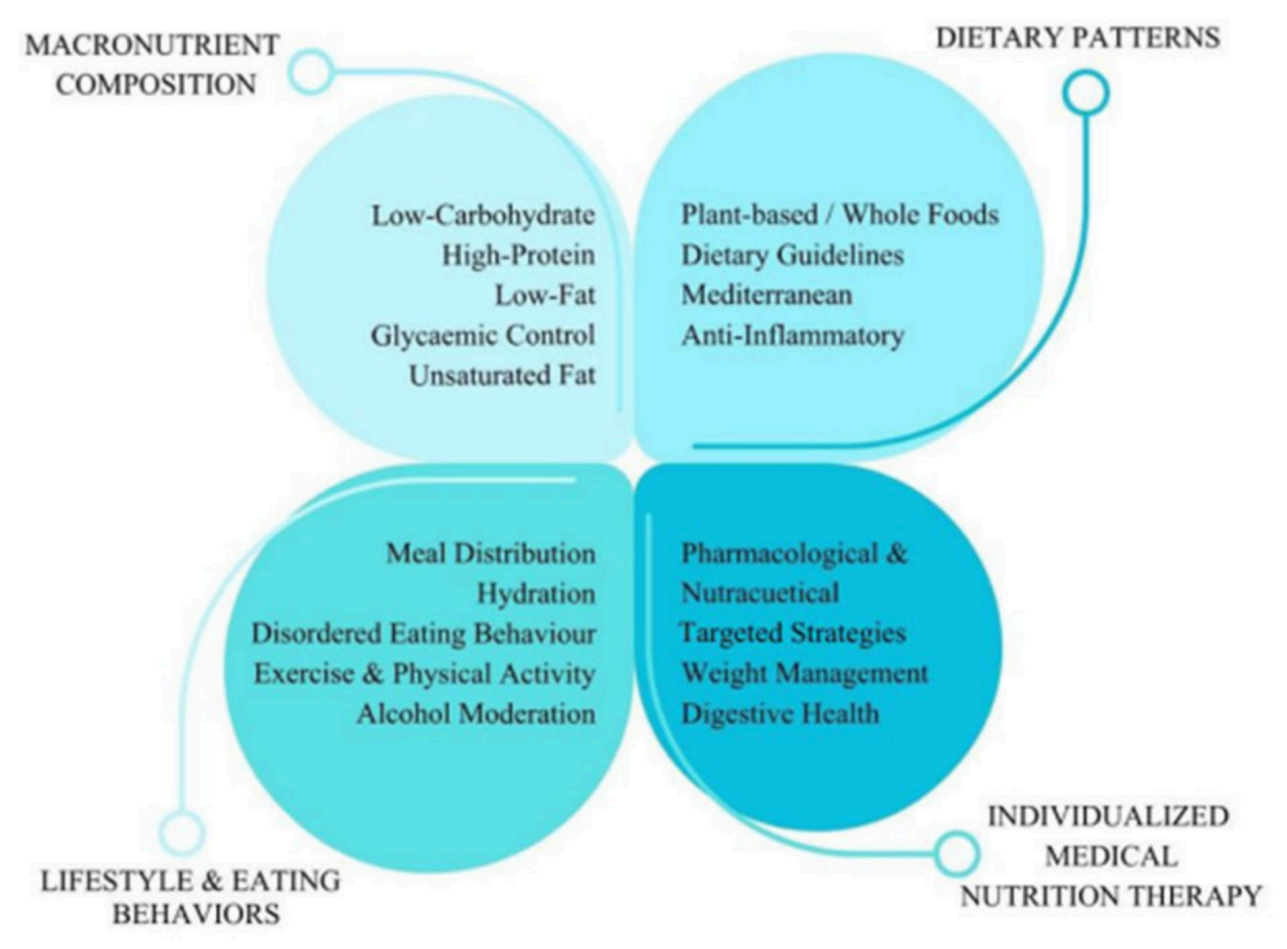
Themes and sub-themes of dietary strategies employed by dietitians for PMOS management.

When asked to identify the most effective dietary approach for managing PMOS, most participants identified more than one approach. Accordingly, frequencies and percentages reported here are based on the total number of responses, rather than the total number of participants. Dietary strategies which dietitians perceived as most effective for managing PMOS were categorized into four overarching themes: *macronutrient composition*, *dietary patterns*, *individualized medical nutrition therapy* and *lifestyle and eating behaviors* (Figure 1).

Manipulation of the macronutrient composition was identified in over 40% of responses (n = 97), with dietitians emphasizing the importance of a low-GI approach (n = 48; 20.3%). Other specific approaches included high-protein diets (n = 19; 8.1%), increased intake of unsaturated fats (n = 14; 5.9%), low-carbohydrate (n = 10; 4.2%) and low-fat diets (n = 6; 2.5%).

Dietary patterns were identified as a key dietary management principle in almost one-third of responses (n = 73; 30.9%). Dietitians most commonly recommended plant-based or whole-food approaches (n = 29, 12.3%) emphasizing a higher intake of wholegrains, fruits, vegetables and legumes. Other frequently cited approaches included those consistent with the Australian Dietary Guidelines (n = 21; 8.9%), a Mediterranean-style diet (n = 18; 7.6%) and anti-inflammatory diets (n = 5; 2.1%).

Almost a quarter of responses identified individualized medical nutrition therapy (n = 42; 17.8%). As a component of this, the use of pharmaceuticals, nutraceuticals and dietary supplements (n = 14; 5.9%), including inositol, vitamin D and iron, was commonly reported amongst dietitians. Respondents also identified patient-centered care, goal setting and supporting behavior change (n = 13; 5.5%) as a key component of medical nutrition therapy. Other targeted strategies included weight management (n = 8; 3.4%) and digestive health (n = 7; 3.0%).

The final theme related to dietary management approaches in practice included lifestyle and eating behaviors (n = 22; 9.3%). Meal distribution (n = 11; 4.7%) was the most frequently reported, which incorporated structured eating patterns, regular meals and consistent meal spacing. Other highlighted strategies included regular exercise (n = 3; 1.3%), adequate hydration (n = 3; 1.3%), and moderation of alcohol intake (n = 2; 0.8%).

Irrespective of the dietary strategies deemed most effective by dietitians, improving PMOS-related symptoms and reducing future cardiometabolic disease risk was the most frequently reported reason as to why such approaches are effective in practice (n = 47; 35.6%). This was followed by weight management and satiety (n = 16; 12.1%) and improved fertility outcomes (n = 16; 12.1%). Dietitians additionally reported that dietary approaches aligning with evidence-based guidelines (n = 14; 10.6%) were also likely to be effective. Less frequently mentioned reasons included holistic well-being (n = 12; 9.1%), improved diet quality (n = 11; 8.3%), adherence and sustainability (n = 7; 5.3%) and improved digestive health (n = 4; 3%).

## 4. Discussion

This study aimed to examine Australian APDs’ knowledge, confidence, practices, and perceived barriers and facilitators in the management of PMOS. Respondents reported moderate knowledge and confidence, with key barriers including challenges in supporting weight management, limited interprofessional collaboration, and dietary misinformation. Facilitators included access to evidence-based approaches, professional development opportunities, and clinical experience. Common management strategies involved macronutrient modification, dietary patterns, individualized nutrition therapy, and lifestyle and eating behavior changes.

Few studies have explored dietetic practice-related barriers in the management of PMOS. In a cross-sectional analysis of dietitians (n = 105) from the United Kingdom, almost two-thirds believed there was insufficient evidence to guide dietary management [17]. Similarly, an Australian study of allied health professionals, including dietitians, reported similar findings consistent with the aforementioned study, despite two iterations of the evidence-based guidelines for the assessment and management of PMOS having been released following this preliminary research [22]. While this suggests an ongoing lack of familiarity with current evidence-based guidelines or difficulties translating recommendations into practice [25,26,27], the present study found 38.4% of dietitians self-reported moderate levels of knowledge, and approximately 40% rated their knowledge as high or very high, suggesting an improving temporal change. To address these persisting gaps in knowledge translation, targeted education and professional development opportunities remain essential. Incorporating PMOS-specific content into tertiary (undergraduate or postgraduate) dietetic curricula could enhance foundational understanding, while undertaking accredited short courses provides necessary ongoing evidence-based professional development and training [28]. Furthermore, establishing accreditation pathways through internationally recognized regulatory bodies could also formalize expertise and provide international applicability. Given that information provision alone is unlikely to elicit meaningful lifestyle change, it remains critical for allied health professionals to strengthen their practical skills in relation to lifestyle management through professional development and knowledge translation. Current evidence-based guidelines emphasize that women should be practically supported to achieve lifestyle changes through behavior management strategies, such as goal setting, problem solving and self-monitoring [11]. This approach has been reinforced by findings from a dietitian-led clinical trial investigating the acceptability and feasibility of a Mediterranean diet (MedDiet) intervention for women with PMOS, which integrated one-on-one dietary counselling and behavior-change strategies [29,30]. In this aforementioned study, a key facilitator of adherence and acceptability was the acquisition of knowledge through tailored educational resources, practical meal-planning suggestions, and clear awareness of MedDiet principles and their associated health benefits [29,30].

Major practice barriers identified in the present study were consistent with previous literature across dietitians [31], allied health [22] and medical professionals [32]. A recurring barrier across all healthcare groups was limited interprofessional collaboration between providers. In the present study, dietitians specifically identified challenges engaging with other multidisciplinary healthcare teams. Consistent with this, Moran et al. [22] also reported low integration of care, including a lack of referrals to allied health professionals delivering lifestyle interventions for PMOS management. Dietitians in the present study also reported few referrals from primary healthcare providers. This aligns with literature on the practice behaviours of GPs, indicating that dietitians receive few referrals for women with PMOS [33,34,35]. Although speculative, a contributing factor may be the confidence of primary healthcare providers in the effectiveness and sustainability of lifestyle approaches for PMOS management. Arasu et al. [32] previously reported that Australian GPs expressed confidence in the provision of lifestyle recommendations for PMOS management. However, this is in contrast to earlier research suggesting that lifestyle education and counselling that is provided is generic [35] and GPs often lack training in supporting long-term behavioral modification. Given the complexity and heterogeneity of PMOS, integrated care involving GPs, endocrinologists, gynecologists, and allied health professionals remains essential [11], alongside strengthened collaboration and clearer role delineation to support lifestyle-focused management of PMOS. Although this model of care is indeed endorsed in the current international evidence-based guidelines [11], access to healthcare services for all women with PMOS remains a limiting factor [13].

Interestingly, in the present study, the most frequently cited barrier was supporting women to achieve weight loss, likely due to women with PMOS having a greater propensity for longitudinal weight gain [11]. Current evidence suggests that many weight loss approaches, including caloric restriction and exercise interventions, show only short-term benefits, with frequent weight regain and high attrition rates observed in both the general population and in women with PMOS [11]. The additional barriers to weight management in PMOS are likely multifactorial, encompassing both psychosocial and physiological factors. Moran et al. [22] also reported that allied health professionals identified psychological comorbidities, including eating disorders, anxiety and depression, as key barriers to lifestyle management. This same study noted that practitioners perceived low motivation among some women with PMOS, which was similarly reported in the present study. These observations underscore the importance of ongoing training and professional development opportunities for dietitians to support and empower women with PMOS in achieving sustainable lifestyle change.

Misinformation emerged as an additional barrier for dietitians, largely stemming from women obtaining nutrition information online and, in some cases, from other healthcare professionals. This aligns with reports that women with PMOS often receive inconsistent lifestyle advice from both medical and allied health professionals [36]. The provision of inconsistent or poor lifestyle support is potentially problematic as it may foster unrealistic expectations, reduced engagement with dietetic care, and prompt women to seek nutrition and dietary information from non-evidence-based sources. Addressing misinformation requires dietitians to not only deliver evidence-based interventions, but also to model critical thinking, actively counter misinformation and establish patient trust [37,38]. Evidence supports that well-structured interventions can mitigate the spread and impact of dietary misinformation [39] through the provision of patient-centered care and empathetic communication, both of which are hallmarks of dietetic practice [40].

Despite fewer than half of dietitians indicating familiarity with the international evidence-based guidelines for PMOS management [11], many reported recommending dietary strategies that were consistent with healthy eating principles, outlined in population-based dietary guidelines. Notably, manipulation of macronutrient content of the diet was among the most frequently reported dietary approaches, despite being less consistent with evidence-based guidelines for PMOS management [11], suggesting a disconnect between the translation of guidelines and clinical practice. Nevertheless, there is evidence, albeit with inconsistent findings, highlighting the potential efficacy of low-glycemic index [41], high-protein [42] and ketogenic diets [43] for insulin resistance, obesity, and anovulation in PMOS management. Irrespective of such evidence, current guidelines emphasize a dietary approach that is consistent with current population-based dietary guidelines and at present, there is no single dietary approach that is superior in supporting improved health-related outcomes for women with PMOS [11]. Nevertheless, several approaches described by dietitians in the present study draw on previously established diabetic-related management principles, including carbohydrate distribution and glycemic control [44], reflecting recognition of insulin resistance as a key pathophysiological feature of PCOS [3,45]. While this diversity highlights flexibility and an individualized approach in dietetic practice, it also underscores a lack of consensus and uniformity in dietary management approaches. In some cases, strategies appeared to prioritize symptom management over broader, long-term metabolic and psychological health outcomes, reinforcing the need for a patient-centered approach whereby women are supported to identify and prioritize their own short- and long-term health goals [13].

In the present study, the application of different dietary approaches was motivated by a range of clinical reasons. Dietitians most frequently selected interventions aimed at symptom management, particularly targeting insulin resistance and glycemic control. Weight management and hormonal regulation were also commonly reported. Less frequently, interventions were chosen because they were evidence-based or focused on holistic patient wellbeing. Collectively, these findings suggest that practice patterns were informed by a combination of clinical reasoning, professional experience, guideline awareness and evidence-based knowledge. Further, this pattern is not unique to PCOS and reflects broader dietetic practice across other clinical areas [46]. Importantly, given the heterogeneity of symptom presentation, tailoring dietary approaches to individualized needs remains critical in PMOS management [11]. For example, although weight loss improves many clinical features of PMOS [11], insulin resistance also affects women within a healthy-weight range [47]. As such, the optimal dietary approach for PMOS remains controversial, with limited high-quality evidence supporting any specific dietary approach beyond population-based guidelines. Therefore, interventions which promote sustainable, long-term behavior change may yield greater improvements in both physical and psychological health [48].

Our findings suggest that despite systemic challenges, many dietitians are effectively using existing literature and resources to inform intervention strategies for women with PMOS. However, current practices remain inconsistent, and professional confidence is often undermined by limited opportunities for continuing education and poor awareness of current guidelines. Strategic dissemination of evidence-based guidelines, including building stronger partnerships [49] alongside the development of structured, PMOS-specific professional development programs by accredited institutions, could help strengthen practice-related confidence and reduce variability in patient care. As such, these strategies have the potential to foster clinical and referral pathways for the dietary and broader management of PMOS through the development of a structured and multidisciplinary framework facilitating the promotion of timely and targeted dietary interventions, including targeted counseling and precision nutrition in order to improve the long-term health outcomes for women with PMOS whilst reducing the impact of systemic barriers.

Despite identifying important practice barriers, this study has several limitations which should be considered when interpreting these findings. Firstly, participant recruitment was likely subject to selection or sampling bias, with dietitians who had a greater interest in PMOS management more likely to complete the questionnaire, resulting in a relatively homogeneous sample. As such, these findings are more likely to represent the views of practitioners with a particular interest in PMOS. As such, future studies could adopt additional qualitative approaches, including focus groups, to better elucidate meaning from individual responses and improve the depth and contextual detail that is acquired from robust qualitative inquiry. Although our sample size was somewhat small and potentially underpowered, the number of respondents in the present study was comparable to previous published research amongst dietitians [17,22]. However, respondents represented a broad range of practice experience; as such, it is likely that clinical experience and exposure are related to knowledge and confidence; however, no formal sub-group analysis was undertaken due to our limited sample size across clinical years of experience. Furthermore, the present study lacked gender and ethnic diversity, exacerbating the homogeneity of our sample. Although efforts were made to recruit participants across the entire country, the sample demonstrated a response bias toward the eastern states of Australia and may not fully reflect the diversity of the national workforce, including regional and remote populations where patient interactions and referral pathways to dietitians are even more limiting. Given the nature of a self-reported online survey, social desirability bias was likely. Moreover, over half of respondents worked in private practice, which may limit the transferability of our findings to other practice settings. Nevertheless, this distribution reflects the current health service structure in Australia, where most dietitians provide outpatient care in private or primary healthcare contexts. The cross-sectional design of the study captured a single point in time, and therefore cannot assess changes in knowledge, confidence and practice over time. Furthermore, given the lack of an existing validated survey instrument, data was captured using an unvalidated tool, thus potentially limiting consistency between studies. In addition, to determine face validity, the questionnaire was only piloted amongst n = 10 Australian dietitians. Although these were representative of the current workforce, it is unlikely that this sample size is adequate prior to participant recruitment. As such, future research should be positioned to further standardize and validate the survey instrument to strengthen future studies. Given that the present study focused exclusively on dietitians, it does not capture the perspectives of other healthcare professionals or system-level factors that may also contribute to barriers in PMOS care and management. This limitation is particularly relevant given the evolving nature of PMOS research and updates to international guidelines and medical terminology. For example, this study was undertaken prior to the renaming of PCOS to PMOS, and as such, may have influenced individual participant responses or even the total response rate if practitioners are unfamiliar with the new terminology, pathophysiology or risk factors of the condition. Although updates in medical nomenclature can indeed be harmful if they cause confusion or misinterpretation, the evolution of the renaming provides an opportunity to update clinical care guidelines, scientific communications and dissemination of education materials to both practitioners and patients, underpinned by more comprehensive research and treatment approaches. The conceptual shift toward recognizing PMOS as a complex metabolic and systemic condition, rather than a reproductive abnormality, allows future nutrition-based research to focus on therapeutic and prescriptive interventions, such as precision nutrition, to individualize dietary recommendations in accordance with genetic, metabolic, hormonal, and lifestyle characteristics of individuals.

### Future Recommendations

This study explored Australian APDs’ knowledge, confidence, practices, and perceived barriers and facilitators toward the dietary management of PMOS. We identified a number of important practice and system-related barriers impeding the provision of dietetic care for women with PMOS. As such, several practice-related priorities have been identified from this research:

1. **Strengthen PMOS-specific dietetic education and professional development**: Dietitians would likely benefit from PMOS-specific content in tertiary curricula, accredited professional development opportunities and improved dissemination of current evidence-based guidelines to strengthen knowledge, confidence and guideline implementation.
2. **Prioritise practical behaviour-change support rather than information provision alone:** Dietetic care should extend beyond nutrition education to include practical behaviour-management strategies including goal setting, problem solving, self-monitoring, tailored resources and practical meal-planning support.
3. **Adopt an individualised, patient-centred approach to dietary management:** Given the heterogeneity of PMOS presentation, dietary interventions should be tailored to individual symptoms, metabolic characteristics, health priorities, preferences and longer-term goals.
4. **Move beyond a narrow focus on weight loss:** Weight management remains an important component of PMOS care; however, clinical practice should recognise that insulin resistance and other metabolic features can occur across the weight spectrum. Management should therefore encompass sustainable lifestyle behaviours and broader metabolic and psychological health outcomes, rather than focusing exclusively on weight reduction.
5. **Improve multidisciplinary collaboration and referral pathways:** More integrated care involving dietitians, GPs, endocrinologists, gynaecologists and other relevant health professionals is needed. Clearer role delineation and routine referral pathways may facilitate timely access to evidence-based dietary and lifestyle interventions.
6. **Improve translation of guidelines into routine clinical practice:** The discrepancy between familiarity with guidelines and reported clinical practices highlights a need for improved knowledge translation and greater consistency in evidence-based dietary management.

## 5. Conclusions

Australian Dietitians reported moderate levels of knowledge and confidence in PMOS management. Supporting women through weight management strategies, limited interprofessional collaboration and dietary misinformation were identified as key practice-related barriers. Limited awareness of the international evidence-based guidelines, combined with limited professional development opportunities and the diversity of individual clinical symptoms and comorbidities, appears to undermine confidence in practice. Although many dietitians apply evidence-based dietary strategies and adapt interventions to symptom presentation, the absence of a holistic, PMOS-specific framework limits the capacity to deliver comprehensive and consistent care. Addressing these gaps through broader dissemination of standardized protocols and guidelines, investment in targeted professional development opportunities (such as online modules and webinars) developed by accredited professionals and societies and establishing interprofessional referral pathways may enhance dietitians’ ability to provide patient-centered, evidence-based dietetic care. Strengthening these areas has the potential to improve health outcomes, patient satisfaction and quality of life for women with PMOS.

## Figures and Tables

**Table 1:** Summary of questions from the online survey **.

No.	Question	Responses
1.	Do you wish to participate in the study?	I have read and understood the above project information. I consent to participate and to have my anonymous data and information used for this project only. I do not wish to participate.
2.	Gender identity	Male Female Non-binary Prefer to self-describe (state below)
3.	What is your age in years?	*Open-ended*
4.	What is your highest level of tertiary qualification?	Diploma Advanced diploma, associate degree Bachelor’s degree Master’s degree Postgraduate degree or doctorate Postgraduate diploma
5.	In what country did you receive your formal Dietetic qualification?	Open-ended
6.	What is your highest level of Dietetic credentialing?	APD AdvAPD Fellow
7.	How many years have you been practising as an APD in Australia?	1–5 years 6–10 years 10–20 years 20+ years
8.	What is your MAIN area of Dietetic practice, e.g., where you spend the majority of your working hours? (tick all that apply)	Community Public health Food Service Clinical or acute care Rehabilitation Private Practice Research Aged Care Education
9.	Have you ever provided Dietetic services or care for a patient/client with PCOS in your area of practice, including research?	Yes No
10.	Have you ever engaged in any professional development opportunities to enhance your knowledge and skills in the area of PCOS management? If yes, please describe these below.	No Yes If yes, please describe these below.
11a.	How would you rate the following? Knowledge in relation to dietary management principles for PCOS?	None/limited Low Moderate High Very High
11b.	How would you rate the following? Confidence in the treatment and management of patients/clients with PCOS?	None/limited Low Moderate High Very High
12.	From a practitioner’s perspective, outline the challenges (if any) you are faced with when treating and managing a patient/client with PCOS?	Open-ended
13.	From a practitioner’s perspective, outline what has made it easier for you to treat and manage patients/clients with PCOS?	Open-ended
	If you are involved in the treatment and management of PCOS as part of your dietetic work, what are the main reason(s) patients/clients engage with your dietetic service? (tick all that apply)	Symptom Management Weight Loss Infertility Cardiovascular Disease Risk Diabetes Risk Psychological Health Disordered Eating I don’t see patients/clients with PCOS
	What do you believe are the main priority treatment areas for women with PCOS? (tick all that apply)	Symptom Management Weight Loss Infertility Cardiovascular Disease Risk Diabetes Risk Psychological Health Disordered Eating I don’t see patients/clients with PCOS
	Are you familiar with best-practice, evidence-based guidelines for lifestyle management of PCOS?	No Yes
	Which dietary management principles do you believe are most effective for the treatment and management of PCOS?	Open-ended
	In line with the question above, why do you believe these are most effective for the treatment and management of PCOS?	Open-ended

** Questions within the survey refer to the term PCOS rather than PMOS. Abbreviations: APD, Accredited Practising Dietitian; AdvAPD, Advanced Accredited Practising Dietitian; PCOS, Polycystic Ovary Syndrome.

**Table 2:** Demographic characteristics of the sample (*n* = 86) **.

Characteristics	
**Gender, *n* (%)**	
Male	3 (3.5)
Female	83 (96.5)
Non-binary	1 (1.2)
Prefer not to say	2 (2.3)
**Age (years)**	
Mean (SD)	37.9 ± 9.9
**Highest level of education, *n* (%)**	
Bachelor’s degree	41 (47.7)
Master’s degree	38 (44.2)
Postgraduate degree or doctorate	5 (5.8)
Postgraduate diploma	2 (2.3)
**Country of qualification, *n* (%)**	
Australia	83 (96.5)
England	1 (1.2)
India	1 (1.2)
South Africa	1 (1.2)
**Dietetic credentials, *n* (%)**	
APD ^a^	84 (97.7)
AdvAPD ^b^	2 (2.3)
**Years practising as APD, *n* (%)**	
1–5 years	31 (36.0)
6–10 years	19 (22.1)
10–20 years	26 (30.2)
20+ years	10 (11.6)
**Main area(s) of practice, *n* (%) ^a^**	
Aged care	2 (1.8)
Clinical or acute care	23 (20.9)
Private practice	51 (46.3)
Education	2 (1.8)
Community	18 (16.4)
Food service	4 (3.6)
Public health	6 (5.5)
Rehabilitation	2 (1.8)
Research	2 (1.8)
**Provided dietetic care for PCOS, *n* (%)**	
No	12 (13.9)
Yes	74 (86.1)
**Engagement in PD for PCOS, *n* (%)**	
No	26 (30.2)
Yes	60 (69.8)
**PD activities related to PCOS, *n* (%) ^b^**	
Webinars	17 (40.5)
Podcasts	3 (7.1)
Self-study	10 (23.8)
Formal courses	8 (19.0)
Seminars/conferences	2 (4.8)
Other PD	2 (4.8)

** Questions within the survey refer to the term PCOS rather than PMOS. ^a^ Participants could select multiple responses. ^b^ Participants could select multiple responses. Abbreviations: APD, Accredited Practising Dietitian; AdvAPD, Advanced Accredited Practising Dietitian; PCOS, Polycystic Ovary Syndrome; PD, Professional Development.

**Table 3:** Barriers and facilitators toward dietetic management of PCOS, as reported by Australian APDs **.

Category	Theme	*n* (% of Responses)	Representative Quotes
**Barriers**	Weight management	24 (20.9)	• Assisting with weight management when client is fatigued and has low motivation. (P7) • Assisting with weight loss when insulin resistance is present. (P18) • There is too much of a focus on weight rather than health. The focus on weight means clients often have disordered eating by the time they come to see me. (P79)
	Interprofessional collaboration	19 (16.5)	• Difficulty engaging client’s care team e.g., GP, gynaecologist, etc. (P23) • Working with GPs who do not have good knowledge of PCOS and therefore do not offer medical support that could be beneficial. (P68) • Medical doctors not referring and a misunderstanding of the importance of early intervention and lifestyle change. (P79)
	Dietary misinformation	14 (12.2)	• Harmful advice from other health professionals and/or social media (e.g., avoid gluten and dairy). (P40) • Lots of competing (?unverified) information online (e.g., keto/paleo for PCOS). (P45) • Limited research around dietary management/influences for PCOS. (P37)
	Individual symptoms and comorbidities	14 (12.2)	• Disparity in symptoms/outcomes. (P78) • Often PCOS is found alongside other comorbidities such as IBS and endometriosis, so a multifaceted approach. (P73) • PCOS is often just one of many issues for an individual when they come in, so it’s the juggle of managing everything at once. (P52)
	Disordered eating behaviours	8 (7.0)	• Biggest challenge I have found is when PCOS clients have eating disorders/disordered eating, but their doctor prioritizes weight loss as the number one solution without proper screening. Many are prescribed weight loss injection medications. (P91) • Higher prevalence of weight concern and eating disorder behaviours (e.g., restriction, binge eating). (P22) • Individual nature of condition, often disordered eating patterns. (P25)
	Lack of knowledge and PD	7 (6.1)	• Not a lot of professional development opportunities available. (P5) • Lack of credentialed courses and professional development opportunities specifically targeting the nutritional management of PCOS. (P76) • Lack of exposure in current area. (P54)
	Patient knowledge and expectations	7 (6.1)	• Expectations of client can be high, e.g., expecting miracles from diet changes/special diets. (P50) • Lack of education around PCOS by clients. (P63) • Clients blaming themselves for symptoms. (P45)
	Patient motivation	6 (5.2)	• Motivating clients to make long-term change to reduce future diet-related disease e.g., T2DM. (P44) • Consistency/engagement. (P41) • Low level of motivation to change in younger clients. (P35)
	Hormonal dysfunction	4 (3.5)	• Role of hormones impacting PCOS. (P34) • Factors related to hormone changes/imbalances. (P31) • Client feeling out of control in their bodies due to impacts of hormone dysregulation. (P22)
	Pharmaceuticals, nutraceuticals and dietary supplements	4 (3.5)	• Dosage of certain things like Omega-3. Unsure if the therapeutic dose is enough. (P6) • Various medications, symptoms and side effects of PCOS and medication. (P15) • Cost of some nutrition supplements that I would recommend. (P80)
	Food beliefs	3 (2.6)	• Restrictive low-carb diet. (P43) • Food and nutrition beliefs. (P56) • Messaging about PCOS in the media has often led to patients believing there is a very specific special diet that will cure their PCOS. (P62)
**Facilitators**	Evidence-based dietary approaches	22 (21.8)	• Applying the evidence from different dietary patterns and layering these into my clients’ diet. (P3) • Availability of clinical guidelines (Monash University, PCOS Guideline) to feel more confident in supporting clients living with PCOS. (P44) • Utilising what best practice evidence we have and utilising a tailored and individualized approach based on clinical judgement. (P37)
	PD opportunities	21 (20.8)	• Eating disorder credentialed. (P63) • Additional self-study, listening to podcasts from PCOS dietitians, and networking with PCOS dietitians has helped my knowledge and skills immensely. (P76) • My additional training in this area through ELNA. (P85)
	Clinical knowledge and experience	20 (19.8)	• Thorough understanding of insulin resistance & glucose control. (P31) • Knowing what questions to ask, how symptoms manifest and the subtypes of PCOS. (P73) • Having specialized knowledge in this area to help clients understand the underlying physiology to link it to how nutrition changes can benefit their condition. (P90)
	Interprofessional collaboration	10 (9.9)	• Liaising with specialist clinicians (incl dietitians) that have training/knowledge of PCOS. (P2) • Supportive families/MDT approach. (P19) • Engagement with client’s care team. (P23)
	Access to patient resources	8 (7.9)	• PEN database. (P24) • Useful tools and resources e.g., Jean Hailes. (P39) • There are some very good sources on the internet/Instagram that young women with PCOS are happy to engage with. (P75)
	Empathy and counselling skills	7 (6.9)	• Counselling/behaviour-change skills. (P11) • Being able to empathise and have a good understanding of the condition on nutrition/lifestyle. (P18) • Understanding how to navigate realistic goal setting and addressing unrealistic expectations raised by social media etc. (P62)
	Behaviour change	6 (5.9)	• Patient’s willingness to change. (P16) • Engaged client willing to engage with behaviour change/advice from dietitian. (P28) • Often, this population group is highly motivated. (P80)
	Pharmaceuticals, nutraceuticals and dietary supplements	2 (2.0)	• Using supplements. (P21) • Supplementation (where necessary) is always well received. (P88)

** Questions within the survey refer to the term PCOS rather than PMOS. Abbreviations: PCOS, Polycystic Ovary Syndrome; APD, Accredited Practising Dietitian; GP, General Practitioner; IBS, Irritable Bowel Syndrome; PD, Professional Development; T2DM, Type 2 Diabetes Mellitus; ELNA, Early Life Nutrition Alliance; MDT, Multidisciplinary Team; PEN, Practice-based Evidence in Nutrition.

## Data Availability

The original contributions presented in the study are included in the article; further inquiries can be directed to the corresponding authors.
